# Comparative evaluation of upper airway dimensions following isolated mandibular setback versus bimaxillary surgery in the surgery-first approach: A 1-year follow-up study

**DOI:** 10.1016/j.jpra.2026.07.032

**Published:** 2026-07-26

**Authors:** Le Tan Hung, Pham Trinh Quoc Khanh, Le Duc Lanh, Tran Ai Khiem, Nguyen My Huyen, Nguyen Trung Hieu

**Affiliations:** a Faculty of Odonto – Stomatology, College of Medicine and Pharmacy of Tra Vinh University, Vinh Long Province, Vietnam; b Can Tho University of Medicine and Pharmacy, Can Tho City, Vietnam; c Faculty of Odonto – Stomatology, Hong Bang International University, Ho Chi Minh City, Vietnam; d Can Tho City General Hospital, Can Tho City, Vietnam

**Keywords:** Surgery-first approach, Mandibular setback, Bimaxillary surgery, Upper airway dimensions, Acoustic pharyngometry, Skeletal class III malocclusion

## Abstract

**Objectives:**

This study aimed to quantitatively compare alterations in upper airway dimensions—specifically volume and cross-sectional area—following isolated mandibular setback versus bimaxillary surgery using the surgery-first approach (SFA) in patients with skeletal Class III malocclusion. To the best of our knowledge, this is the first study to directly compare these two surgical protocols exclusively under the SFA paradigm with acoustic pharyngometry and per-millimetre analysis over a 1-year follow-up.

**Materials and methods:**

A retrospective cohort investigation was conducted on 40 skeletally mature Class III adults, equally allocated into two cohorts: Group 1 underwent isolated mandibular setback via bilateral sagittal split osteotomy (BSSO, n=20), while Group 2 received bimaxillary orthognathic surgery (maxillary advancement combined with mandibular setback, n=20). Upper airway parameters were objectively assessed using acoustic pharyngometry at three distinct intervals: one week preoperatively (T0), one month postoperatively (T1), and one year postoperatively (T2). Spatial airway variations were quantified as percentage changes, with the magnitude of airway reduction and subsequent recovery calculated per millimetre (mm) of surgical mandibular setback.

**Results:**

At T1, both surgical interventions resulted in a statistically significant reduction in airway dimensions. Nevertheless, the extent of airway constriction was markedly more severe in Group 1 compared to Group 2, affecting both volume (-22.84% vs. -7.96%; p < 0.01) and cross-sectional area (-21.45% vs. -6.79%; p < 0.01). The volumetric reduction per millimetre of mandibular setback in Group 1 was -1.168 cc/mm, which was substantially greater than the -0.458 cc/mm observed in Group 2 (p < 0.01). By T2, a distinct airway recovery (relapse) was documented; Group 1 exhibited a higher volumetric recovery rate (+15.83%) than Group 2 (+4.63%). Despite this rebound, the overall airway dimensions at the one-year follow-up (T2) remained significantly larger and better preserved in the bimaxillary cohort than in the isolated setback group (p < 0.05).

**Conclusions:**

Performing an isolated mandibular setback under the SFA protocol induces considerable upper airway constriction. Conversely, incorporating maxillary advancement via bimaxillary orthognathic surgery significantly preserves airway dimensions. Consequently, bimaxillary intervention should be considered a safer surgical indication to proactively mitigate the risk of respiratory impairment, particularly in Class III patients requiring substantial mandibular retropositioning. This study provides new evidence that bimaxillary surgery better preserves airway dimensions in SFA compared with isolated mandibular setback.

## Introduction

Orthognathic surgery (OGS) combined with orthodontic treatment is widely used for correcting severe skeletal Class III malocclusions, simultaneously restoring masticatory function and facial harmony.^1^^,^^2^ The conventional orthognathic approach (COA) has been employed to achieve presurgical dental decompensation and a stable postoperative occlusion, but is associated with prolonged treatment duration and a transient yet marked deterioration of facial aesthetics during the presurgical phase.^1^ In recent years, the surgery-first orthognathic approach (SFA) has been proposed as an alternative.^3^ By bypassing presurgical orthodontic preparation, the SFA shortens overall treatment duration—capitalising on the regional acceleratory phenomenon (RAP)—while providing immediate aesthetic gratification for the patient.^1^^,^^4^^,^^5^

Despite these advantages, the impact of the SFA on upper airway dimensions remains a critical concern. Mandibular setback—a common surgical option for Class III prognathism—alters the hyoid bone's position and displaces the tongue base posteriorly, thereby inducing notable constriction and narrowing of the posterior airway space (PAS).^6^, ^7^, ^8^, ^9^ The resulting reduction in total volume and minimal cross-sectional area (CSAmin) within the oropharyngeal and hypopharyngeal compartments serves as a latent predisposing factor for obstructive sleep apnea (OSA).^6^^,^^10^^,^^11^ Because presurgical dental decompensation is omitted in SFA, a larger mandibular setback and greater clockwise mandibular rotation may be required to achieve transitional occlusion.^10^^,^^12^^,^^13^ Agarwal et al. (2020) corroborated this by demonstrating that an SFA-driven mandibular setback precipitates a more drastic immediate postoperative decline in airway volume (a reduction of 1.06 cc per millimetre of setback, contrasting with 0.56 cc/mm in the COA), with a significantly higher rate of airway rebound at the one-year follow-up, primarily attributed to initial occlusal instability.^4^ Compounding this issue, postoperative kinematic adaptations of the tongue—specifically decreased tongue length and increased tongue height—show an inverse correlation with the PAS, further exacerbating the risk of airway compromise.^14^^,^^15^

To mitigate the risk of postoperative airway compromise and OSA, bimaxillary surgery combining maxillary advancement with mandibular setback has been increasingly advocated.^2^^,^^16^ Maxillary advancement effectively stretches the palatal and pharyngeal soft-tissue musculature, thereby counteracting the airway constriction induced by mandibular setback. Extensive literature on COA cohorts has shown that bimaxillary interventions provide a safer airway profile than isolated single-jaw procedures.^2^^,^^9^^,^^17^^,^^18^ However, there is currently a paucity of comprehensive quantitative investigations evaluating volumetric and cross-sectional spatial parameters and directly comparing airway changes between isolated single-jaw and bimaxillary surgeries performed exclusively under the SFA paradigm. Additionally, despite established physiological disparities in baseline airway dimensions between males and females, gender-specific response and recovery trajectories of the upper airway following SFA interventions remain underexplored.^2^^,^^15^

In terms of methodology, traditional upper airway evaluations predominantly rely on two-dimensional (2D) lateral cephalograms, which fail to capture transverse dimensions and volumetric changes.^19^^,^^20^ While three-dimensional (3D) cone-beam computed tomography (CBCT) provides excellent spatial resolution, it involves radiation exposure, rendering it less suitable for repeated short-interval postoperative monitoring. Alternatively, acoustic pharyngometry (AP) demonstrates reproducibility and a strong correlation with CBCT volumetric analyses (r ranging from 0.75 to 0.94). This robust correlation validates AP as a highly dependable, radiation-free complementary tool for continuous, dynamic airway monitoring, effectively overcoming the static and radiological limitations inherent to CBCT.^20^, ^21^, ^22^ By utilising the physical principle of acoustic reflection, AP can non-invasively and rapidly quantify pharyngeal volume and minimal cross-sectional area (mCSA) in a dynamic state.^23^^,^^24^ The mCSA parameter derived from AP has been validated as a significant independent predictor for the presence and severity of OSA.^8^ Because it is entirely free of ionising radiation, AP also safely permits continuous monitoring and real-time dynamic evaluation of airway collapsibility throughout various treatment phases.^8^^,^^25^

To date, limited literature has directly compared upper airway changes between isolated mandibular setback and bimaxillary surgery within the SFA protocol using acoustic pharyngometry. Standardising airway alterations per millimetre of mandibular setback may also allow more clinically relevant comparisons between surgical movements. Therefore, this study aimed to compare short-term and 1-year postoperative changes in upper airway volume and minimal cross-sectional area between these two surgical approaches in patients managed with the SFA, and to evaluate the relative impact of each modality per millimetre of mandibular setback.

## Materials and methods

### Study design and cohort selection

This retrospective cohort study utilised archival data from 40 individuals who underwent orthognathic correction. The primary focus was to evaluate airway dimensional changes by stratifying subjects into two surgical protocols: single-jaw surgery (isolated mandibular setback) versus bimaxillary orthognathic surgery. Upper airway dimensions (cross-sectional area and volume) were objectively quantified using acoustic pharyngometry (Eccovision Acoustic Pharyngometer). All pharyngometric recordings were executed by a single calibrated examiner adhering to a standardised protocol. The magnitude of surgical skeletal displacement was extracted from postoperative cephalometric analyses and surgical records.

### Eligibility criteria

Participants were recruited based on the following inclusion parameters:

• Chronological age between 18 and 30 years, with an equal gender distribution.
• Availability of comprehensive medical, surgical, and orthodontic records.
• Confirmed skeletal maturity presenting with a Class III skeletal pattern (ANB < −2°) and a reverse overjet exceeding 2 mm.
• Excellent general health, with no systemic morbidities, bone metabolic disorders, or contraindications for general anaesthesia.
• Orthodontic management via a non-extraction philosophy, with a tooth-size arch-length discrepancy of 5 mm or less.

Candidates were excluded if they presented with:

• A history of prior maxillofacial trauma, earlier orthognathic procedures, or interceptive craniofacial treatments.
• Obese body mass index (BMI ≥ 30.0 kg/m²).
• Any pre-existing compromised airway condition, including but not limited to obstructive respiratory disorders, severe asthma, deviated nasal septum, macroglossia, or hypertrophic tonsils/adenoids.

### Sample stratification and treatment protocol

Patients were divided into two groups with equal gender distribution to avoid gender bias:

• Group 1: Single-jaw surgery (isolated mandibular setback by BSSO) under the SFA.
• Group 2: Bimaxillary surgery (maxillary advancement combined with mandibular setback) under the SFA.

**Fig. 1:**
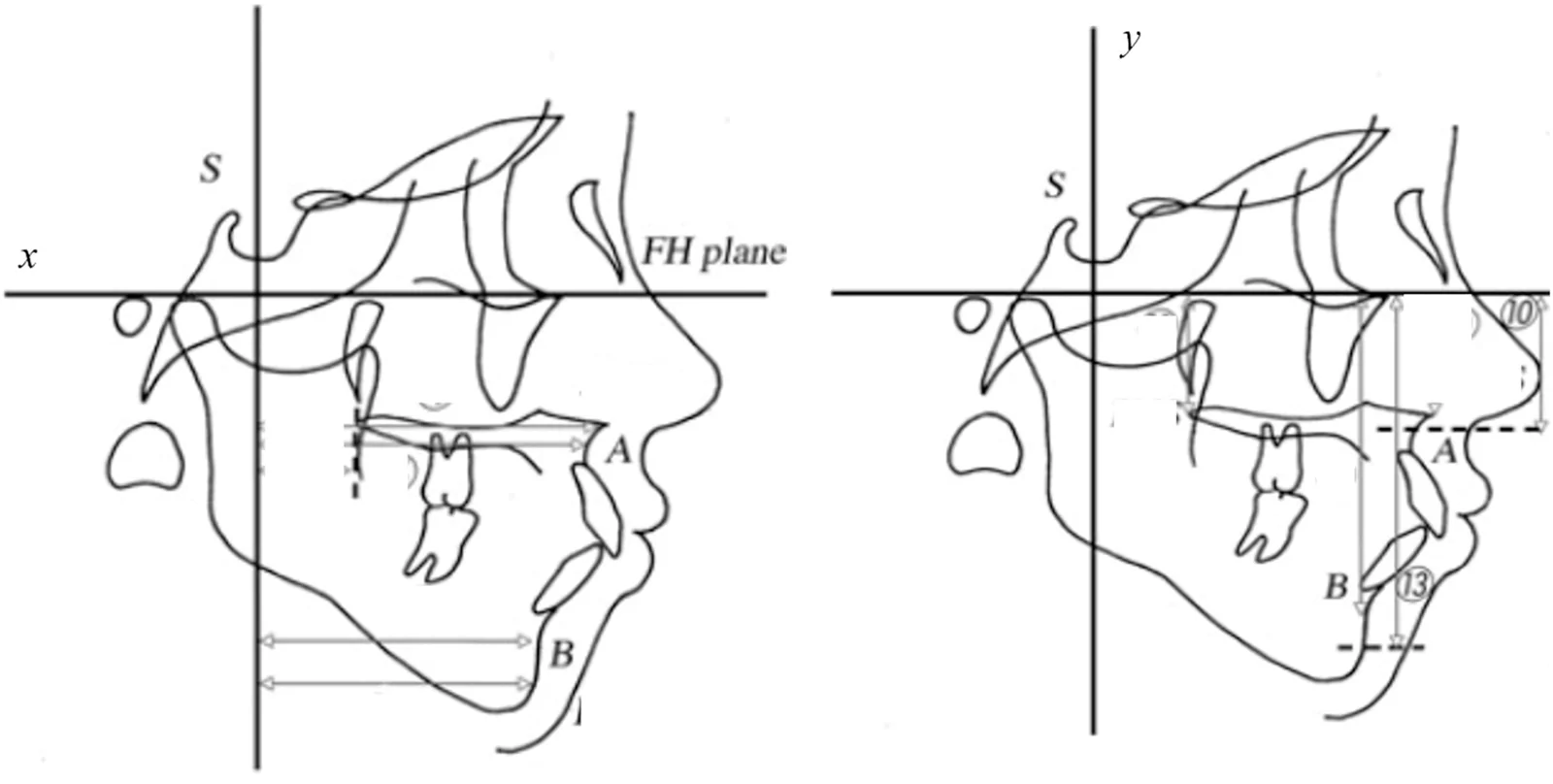
Measuring the distance from the A-point (maxillary) and B-point to the X and Y axes to access the horizontal (sagittal) and vertical movements.

To quantitatively evaluate the magnitude and direction of surgical skeletal movements (Point A for the maxilla and Point B for the mandible), a specific Cartesian coordinate system was established on lateral cephalograms. The horizontal reference plane (X-axis) was defined by the Frankfort horizontal (FH) plane. The vertical reference plane (Y-axis) was defined as a line passing through the Sella (S) point and oriented perpendicular to the FH plane. The displacements of Point A and Point B along these axes were recorded to calculate the horizontal (sagittal) and vertical movements (Fig. 1).

**Fig. 2:**
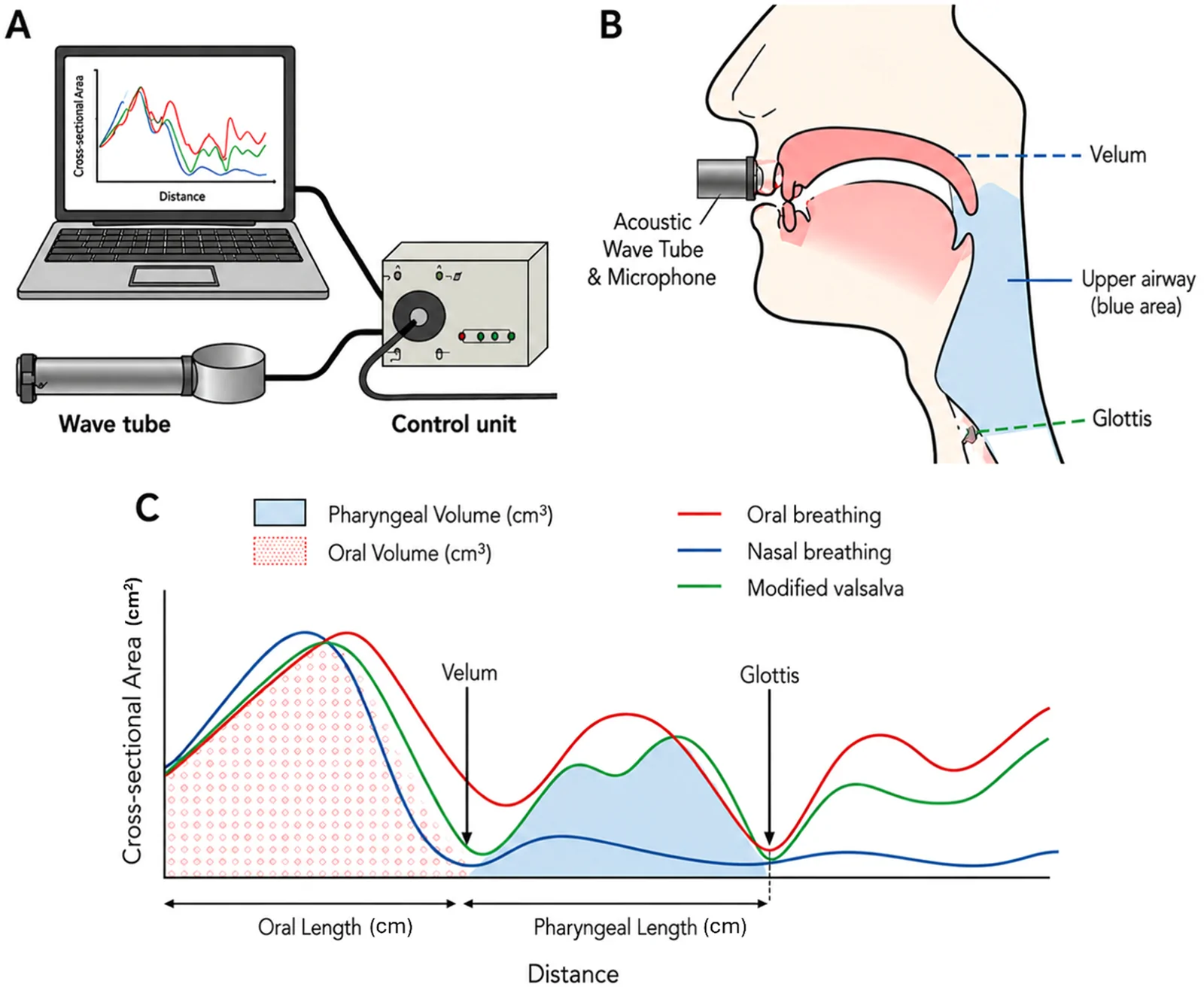
Acoustic Pharyngometry (AP) system and principles of upper airway evaluation .^36^ (A) Components of the Acoustic Pharyngometry setup. (B) Anatomical boundaries of the upper airway (blue area) (C) Schematic representation of the AP setup and the three breathing tasks performed. The blue-shaded region under the Pharyngogram represents the total upper airway volume, derived from the cross-sectional area as a function of the pharyngeal length.

Upper airway parameters were objectively assessed using acoustic pharyngometry (Eccovision Acoustic Pharyngometer) (Fig. 2). The proportional variance (percentage change) in airway dimensions was calculated between T0 (one week before surgery) and T1 (one month after surgery) to appraise the immediate surgical impact, and between T1 and T2 (one year after surgery) to assess long-term airway stability or relapse. Volumetric and cross-sectional alterations, along with the corresponding relapse rates, were further quantified as ratios per millimetre of mandibular setback to facilitate precise inter-group comparisons. The amount of mandibular setback (in mm) was extracted from treatment records.

Airway changes per millimetre of mandibular setback were calculated as:

• Percentage (%) change: between T1 and T0 (postoperative narrowing) and between T2 and T1 (relapse/recovery).
• Airway reduction per mm of setback at T1 = (T1 − T0) / setback (mm).
• Airway recovery (relapse) per mm of setback at T2 = (T2 − T1) / setback (mm).

To eliminate operator and biomechanical confounding variables, all surgical procedures were executed under general anaesthesia by the identical maxillofacial team. Furthermore, to eliminate body mass index (BMI) as a confounding factor for airway constriction and OSA risk, obese subjects were strictly excluded from the study. Presurgical orthodontic preparation uniformly utilised a 0.022-inch slot MBT pre-adjusted edgewise bracket system with a standardised archwire sequence. Postoperatively, the surgical splint was maintained to guide the interim transitional occlusion and was subsequently removed 2 to 4 weeks after surgery. Following splint removal, the active postsurgical orthodontic phase commenced immediately to capitalise on the regional acceleratory phenomenon (RAP).

To control for measurement bias, all lateral cephalograms were digitised and analysed by a single experienced orthodontist who was strictly blinded to patient demographic information and the specific evaluation time points. The resulting Intraclass Correlation Coefficient demonstrated excellent measurement agreement (ICC > 0.90 for all cephalometric variables) in a selected subset of 20 cephalograms re-measured by the same investigator after a two-week washout period. Similarly, all acoustic pharyngometry assessments were executed by a single calibrated clinician with specialised expertise in AP instrumentation and the standard operating protocol. To further ensure objectivity, the examiner was also blinded to the primary research objectives and to the specific surgical modality assigned to each subject.

### Statistical methodology

Data were aggregated in Microsoft Excel. Categorical variables are reported as frequencies (n); continuous measurements as means ± SD. After confirming data normality, inter-cohort comparisons used the independent-samples Student's t-test, and longitudinal intra-cohort changes across the three time points were assessed by repeated-measures ANOVA. All tests were two-tailed with significance at p < 0.05. Analyses were conducted using SPSS v22.0 (IBM Corp., Armonk, NY, USA).

## Results

This study quantitatively evaluated dimensional alterations of the upper airway—mean volume and cross-sectional area—between two SFA cohorts: Group 1 (isolated mandibular setback) and Group 2 (bimaxillary orthognathic surgery). Data were acquired one week preoperatively (T0), one month postoperatively (T1), and one year postoperatively (T2).

Table 1 summarises the baseline demographic data, preoperative cephalometric parameters, and the magnitude of surgical movements for both cohorts. The population was matched for gender (10 males and 10 females per group). There were no significant differences in mean age (23.45 ± 3.12 vs. 24.10 ± 2.85 years; p = 0.49) or BMI (21.56 ± 1.80 vs. 22.09 ± 2.14 kg/m²; p = 0.32). Both groups exhibited severe skeletal Class III discrepancy preoperatively, with comparable ANB angles (p = 0.06).

**Table 1:** Baseline demographics, skeletal characteristics and surgical movements of the study population.

*Variables*	*Group 1 (n=20)*	*Group 2 (n=20)*	*p-value*
***Demographics***			
***Age (years),****Mean ± SD*	*23.45 ± 3.12*	*24.10 ± 2.85*	*0.49^(a)^*
***Gender (Male/Female),****n*	*10 / 10*	*10 / 10*	*1.00^(b)^*
***BMI (kg/m^2^),****Mean ± SD*	*21.56 ± 1.80*	*22.09 ± 2.14*	*0.32^(a)^*
***Preoperative Skeletal Baseline***			
***SNA (^o^),****Mean ± SD*	*81.26 ± 3.10*	*79.80 ± 2.91*	*0.13^(a)^*
***SNB (^o^),****Mean ± SD*	*86.10 ± 2.85*	*85.45 ± 3.20*	*0.49^(a)^*
***ANB (^o^),****Mean ± SD*	*-4.85 ± 1.20*	*-5.65 ± 1.45*	*0.06^(a)^*
***Surgical Movements (T1-T0)***			
***Maxilla (Point A)***			
*Sagittal advancement (mm)*	*-*	*4.32 ± 1.15*	*< 0.001^(c)^*
*Vertical impaction/extrusion (mm)*	*-*	*1.20 ± 0.85*	*< 0.001^(c)^*
***Mandible (Point B)***			
*Sagittal setback (mm)*	*-5.92 ± 1.45*	*-5.10 ± 1.30*	*0.07^(a)^*
*Vertical movement (mm)*	*0.85 ± 0.61*	*1.04 ± 0.75*	*0.35^(a)^*
***Rotational Changes***			
*Δ SN-MP (^o^)*	*2.15 ± 1.43*	*1.50 ±1.22*	*0.13^(a)^*

Group 1: Isolated mandibular setback; Group 2: Bimaxillary surgery (Maxillary advancement + Mandibular setback); BMI: Body Mass Index; SN-MP: Sella-Nasion to Mandibular Plane angle. Positive values in rotational changes indicate clockwise rotation (CWR). ^(a)^ Independent Samples t-test; ^(b)^ Chi-square test; ^(c)^ Mann-Whitney U test. p < 0.05 indicates statistical significance.

Group 2 underwent a mean maxillary sagittal advancement of 4.32 ± 1.15 mm. The mandibular sagittal setback at Point B was slightly greater in the single-jaw group (−5.92 ± 1.45 mm) than in the bimaxillary group (−5.10 ± 1.37 mm), though this difference marginally missed statistical significance (p = 0.07). Both protocols required a postoperative clockwise mandibular rotation to establish an interim transitional occlusion, reflected by an increase in the SN-MP angle (+2.15 ± 1.43° for Group 1 and +1.50 ± 1.22° for Group 2; p = 0.13) (Tables 2 and 3).

**Table 2:** Comparison of mean airway volume (cc).

Volume (cc)	Group 1 (n-20)	Group 2 (n-20)	P-value (Inter-group)
**T0** (Mean ± SD)	29.87 ± 4.59 cc	31.00 ± 4.54 cc	*0.4386*
**T1** (Mean ± SD)	22.78 ± 4.73 cc	28.10 ± 3.82 cc	*0.0004*⁎⁎
**T2** (Mean ± SD)	25.66 ± 4.52 cc	29.05 ± 4.11 cc	0.0176*
**% Change at T1 from T0**	-22.84%	-7.96%	*0.0046*⁎⁎
**% Change at T2 from T1** (Relapse)	+15.83%	+4.63%	*0.1082*
**Airway change per mm setback at T1 from T0**	-1.168 cc/mm	-0.458 cc/mm	*0.0067*⁎⁎
**Airway change (relapse) per mm setback at T2 from T1**	+0.453 cc/mm	+0.156 cc/mm	*0.2623*
* P-value (Intra-group)*			
* T0 versus T1*	*< 0.0001*⁎⁎	0.0112*	
* T1 versus T2*	0.0296*	*0.3771*	

Values are mean ± SD; P-value (inter-group) by the independent-samples t-test. P-value (intragroup) by repeated-measures ANOVA.

⁎ P-value < 0.05,

⁎⁎ P-value < 0.01.

**Table 3:** Comparison of mean cross-sectional area (cm²).

Mean Cross-Sectional Area (cm²)	Group 1 (n-20)	Group 2 (n-20)	P-value (Inter-group)
***T0****(Mean ± SD)*	*3.08 ± 0.50 cm²*	*3.10 ± 0.48 cm²*	*0.8980*
***T1****(Mean ± SD)*	*2.38 ± 0.47 cm²*	*2.85 ± 0.40 cm²*	*0.0016*⁎⁎
***T2****(Mean ± SD)*	*2.59 ± 0.49 cm²*	*2.95 ± 0.42 cm²*	0.0171*
***% Change at T1 from T0***	*-21.45%*	*-6.79%*	*0.0060*⁎⁎
***% Change at T2 from T1 (Relapse)***	*+12.08%*	*+4.92%*	*0.3291*
***Δ T1-T0 Airway change per mm setback***	*-0.109 cm²/mm*	*-0.038 cm²/mm*	*0.0075*⁎⁎
***Δ T2-T1 Airway change (relapse) per mm setback***	*+0.030 cm²/mm*	*+0.016 cm²/mm*	*0.5822*
*P-value (Intra-group)*			
*T0 versus T1*	*< 0.0001*⁎⁎	0.0331*	
*T1 versus T2*	*0.1148*	*0.3684*	

Values are mean ± SD; P-value (inter-group) by the independent-samples t-test. P-value (intragroup) by repeated-measures ANOVA.

⁎ P-value < 0.05,

⁎⁎ P-value < 0.01.

### Alterations in mean airway volume

At baseline (T0), mean airway volumes showed no significant intergroup difference (Group 1: 29.87 ± 4.59 cc; Group 2: 31.00 ± 4.54 cc; p > 0.05). Intragroup analyses at T1 revealed substantial volumetric reduction in both groups (p < 0.0001 for Group 1; p = 0.0112 for Group 2).

Intergroup comparison showed that this postoperative decline was significantly more profound in Group 1 (T1: 22.78 ± 4.73 cc vs. 28.10 ± 3.82 cc; p = 0.0004). Group 1 experienced a volume reduction of 22.84%, markedly higher than the 7.96% in Group 2 (p = 0.0046). The discrepancy was even more pronounced when standardised by surgical movement: 1.168 cc/mm of mandibular setback in Group 1 versus 0.458 cc/mm in Group 2 (p = 0.0067).

At T2, a general trajectory of airway recovery was observed. Intragroup evaluation indicated a statistically significant volumetric increase from T1 to T2 within Group 1 (p < 0.05), whereas the corresponding anatomical rebound in Group 2 lacked statistical significance (p > 0.05). Group 1 demonstrated a volumetric recovery (relapse) rate of +15.83% (equivalent to +0.453 cc/mm of setback), exceeding the +4.63% (+0.156 cc/mm of setback) noted in Group 2. Nonetheless, the intergroup variance in the magnitude of this recovery did not reach statistical significance (p > 0.05). Ultimately, at the T2 interval the mean airway volume in Group 2 (29.05 ± 4.11 cc) remained significantly larger and better preserved than that of Group 1 (25.66 ± 4.52 cc).

### Alterations in mean cross-sectional area

Mirroring the volumetric trends, baseline (T0) mean cross-sectional areas were comparable between groups (Group 1: 3.08 ± 0.50 cm²; Group 2: 3.10 ± 0.48 cm²).

At T1, both groups showed a significant reduction in airway area (p < 0.0001 for Group 1; p = 0.0331 for Group 2). The cross-sectional area at T1 was significantly larger in Group 2 (2.85 ± 0.40 cm²) than in Group 1 (2.38 ± 0.47 cm²). The proportional area constriction reached 21.45% in Group 1 versus 6.79% in Group 2. Standardised per millimetre of mandibular setback, the decrement was −0.109 cm²/mm in Group 1 and −0.038 cm²/mm in Group 2 (p < 0.05).

At T2, both cohorts exhibited a marginal rebound that did not reach statistical significance. Relative recovery was +12.08% (+0.030 cm²/mm) in Group 1 and +4.92% (+0.016 cm²/mm) in Group 2; the intergroup difference was not significant (p > 0.05). At T2, the mean cross-sectional area in the bimaxillary cohort (2.95 ± 0.42 cm²) remained significantly greater than in the isolated setback cohort (2.59 ± 0.49 cm²; p = 0.0171).

## Discussion

The primary objective of this study was to quantitatively compare alterations in upper airway dimensions (volume and cross-sectional area) following isolated mandibular setback versus bimaxillary surgery managed under the SFA protocol. Both modalities precipitated a significant constriction in airway volume and area immediately postoperatively (T1); however, the magnitude was markedly greater in the single-jaw cohort. At the 1-year follow-up (T2), although the isolated setback group showed greater anatomical recovery (relapse), overall airway dimensions were ultimately better preserved by the bimaxillary intervention.

The proportional recovery of airway dimensions at the one-year postoperative mark (T2) in our cohort occurred at a higher rate than has been reported for COA cohorts. These findings may be interpreted within the specific biomechanical context of the SFA, which differs from the COA. In COA, presurgical orthodontic decompensation facilitates a stable postoperative occlusion and limits the magnitude of the required surgical setback.^4^^,^^26^ Conversely, SFA bypasses presurgical orthodontic decompensation; it frequently requires larger surgical setbacks and complex rotational movements to achieve an interim transitional occlusion (ITO). This ITO, combined with the regional acceleratory phenomenon (RAP), contributes to accelerated skeletal remodelling and subsequent neuromuscular adaptation.^1^^,^^4^^,^^27^ Consequently, SFA patients exhibit distinct patterns of airway rebound and skeletal relapse compared with those undergoing COA. Kongsong et al. (2025) corroborated this by demonstrating that SFA patients typically undergo more extensive mandibular retropositioning and rotation than COA patients, leading to distinct physiological differences in airway rebound: SFA leads to postoperative elongation of the mCSA of 0.034 cc/mm of setback, whereas COA presents 0.016 cc/mm at the one-year mark.^15^ Consistent with a previous CBCT-based study (Hussain et al., 2025), isolated single-jaw surgery decreases both total pharyngeal volume and minimum cross-sectional area, whereas bimaxillary surgery mitigates this decrease.^7^^,^^15^^,^^18^ This is attributed to the posterior repositioning of the mandible, which retrodisplaces the tongue base and hyoid bone into the pharyngeal space. Conversely, the maxillary advancement in bimaxillary surgery translates the soft palate and pharyngeal musculature anteriorly, generating compensatory tension that counteracts the soft-tissue compression induced by the mandibular setback.^28^, ^29^, ^30^

The initial postoperative trend of airway constriction in both cohorts aligns with global findings; however, the quantitative magnitude differs from studies relying on 2D lateral cephalometry, such as Kori et al. (2022),^6^ Sahoo et al. (2020),^7^ and Thapa et al. (2023),^17^ because 2D cephalometry is limited by anatomical superimposition.^31^ Tsolakis et al. (2016) demonstrated a remarkably strong correlation between AP and CBCT pharyngeal measurements, with a discrepancy of less than 4% in mCSA and approximately 758 mm³ in pharyngeal volume—clinically insignificant.^22^ Similarly, D'Urzo et al. reported that mCSA measured by acoustic reflection and CT exhibited a very low discrepancy (<4.3%) with an excellent correlation coefficient (r = 0.92).^21^ Although CBCT and MRI remain reference modalities for high-resolution 3D anatomical mapping, they provide only a static representation of a dynamic physiological structure; cumulative radiation exposure and cost restrict their use for repeated short-interval monitoring.^4^ AP offers a dynamic, radiation-free, cost-effective alternative for assessing airway collapsibility; its derived mCSA is an established predictor of OSA severity and demonstrates high test-retest reliability (intra-class correlation r > 0.77).^4^^,^^20^^,^^22^

In terms of airway evaluation modalities, although acoustic pharyngometry (AP) is utilized for its non-invasive capabilities, it cannot provide direct anatomical mapping or localize specific soft-tissue impingements. It cannot precisely pinpoint whether airway narrowing is caused by specific soft-tissue factors (e.g., enlarged tonsils, altered tongue posture, hyoid bone displacement) or by neuromuscular compensation.^4^^,^^20^ Therefore, it is important to acknowledge the emerging role of Magnetic Resonance Imaging (MRI), particularly dynamic MRI. Unlike AP, which provides a one-dimensional acoustic reflection profile of the cross-sectional area and volume, dynamic MRI allows for a comprehensive, real-time, three-dimensional visualization of soft-tissue dynamics during the respiratory cycle.^7^ Recent literature highlights the superiority of dynamic MRI in assessing precise anatomical adaptations, such as tongue deformation patterns and pharyngeal wall collapsibility, thereby identifying specific anatomic risk factors for sleep-disordered breathing without exposing the patient to ionizing radiation .^7^^,^^20^ However, the routine clinical application of dynamic MRI for post-orthognathic follow-up is currently hindered by its high cost, time-consuming nature, limited accessibility, and potential patient intolerance to the supine position due to claustrophobia. AP, therefore, remains a highly practical and reliable alternative for longitudinal volumetric screening in the outpatient orthognathic setting. Future studies integrating the dynamic functional information provided by MRI, the high-resolution three-dimensional anatomical detail offered by CBCT (which is more affordable and remains indispensable for precise presurgical planning and postoperative assessment), and the accessibility of AP to provide a more comprehensive understanding of post-surgical airway adaptations.

Regarding the correlation between morphology and function, the observed physical constriction of the airway space does not necessarily translate into the onset of obstructive sleep apnea syndrome (OSAS).^15^ Ronchi et al. (2023) concluded that there is no direct causal relationship between mandibular setback surgery (particularly with displacements < 8 mm) and the genesis of OSAS, provided the patient does not possess underlying risk factors such as obesity (high BMI).^32^ Parameters such as the Apnea–Hypopnea Index (AHI) and Oxygen Desaturation Index (ODI) may exhibit transient elevations during the first postoperative month secondary to soft-tissue oedema, but generally improve and normalise after 6 months. This divergence between morphological parameters (dimensional narrowing) and physiological function (absence of OSAS) is rationalised by the adaptive capacity of the pharyngeal neuromuscular system, which increases resting muscle tone to maintain airway patency during sleep.^33^, ^34^, ^35^

By standardising volumetric airway decrement per millimetre of setback, this study provides a metric with comparable clinical predictive value across surgical interventions. For skeletal Class III patients presenting with a preoperatively narrow airway or requiring substantial mandibular retropositioning under the SFA, bimaxillary surgery represents a safer surgical indication, proactively maintaining airway volume and preventing structural alterations from exceeding the patient's physiological compensatory threshold.

Although AP does not provide intuitive three-dimensional anatomical visualisation equivalent to CBCT, it is anticipated that future prospective studies strictly isolating variables specific to the SFA protocol—such as completion of postoperative orthodontic treatment at T2, and postoperative changes in BMI and skeletal stability—and integrating 3D imaging (CBCT/MRI) with functional polysomnographic metrics over long-term follow-up will establish a more comprehensive assessment of both the static anatomical characteristics and the dynamic functional behaviour of the upper airway in skeletal Class III patients and other dentofacial deformities undergoing SFA orthognathic surgery.

## Conclusion

Performing an isolated mandibular setback under the SFA induces considerable upper airway constriction, whereas bimaxillary orthognathic surgery significantly preserves airway dimensions. Bimaxillary intervention should therefore be considered a safer surgical indication to proactively mitigate the risk of respiratory impairment, particularly in Class III patients requiring substantial mandibular retropositioning. This study provides new evidence that bimaxillary surgery better preserves airway dimensions under the SFA compared with isolated mandibular setback.

## Sources of support in the form of grants

None

## Ethical approval

This study was supported by the Independent Ethics Committee IEC, Tra Vinh University (Ref number 185/GCN.ĐC-HĐĐĐ, signed August 25^th^, 2025).

## Funding

None

## Patient consent

Obtained.

## Declaration of competing interest

None declared
